# Radon Exposure Assessment and Relative Effective Dose Estimation to Inhabitants of Puglia Region, South Italy

**DOI:** 10.3390/ijerph121114948

**Published:** 2015-11-23

**Authors:** Maria Quarto, Mariagabriella Pugliese, Giuseppe La Verde, Filomena Loffredo, Vincenzo Roca

**Affiliations:** 1 Dipartimento di Fisica, Università di Napoli Federico II, Naples 80126, Italy; E-Mails: pugliese@na.infn.it (M.P.); giuseppelaverde@live.it (G.L.V.); roca@na.infn.it (V.R.); 2 Istituto Nazionale di Fisica Nucleare (INFN), Sezione di Napoli, Naples, 80126, Italy; E-Mail: filomena.loffredo@unina.it

**Keywords:** radon indoor, LR-115 detectors, effective dose, Puglia-South Italy

## Abstract

Indoor radon concentrations were measured in dwellings of the Puglia region in Southern Italy using LR-115 passive detectors. The results show that the radon concentrations varied from 15 ± 2 to 2166 ± 133 Bq/m^3^ with a geometric mean of 114 Bq/m^3^ and a geometric standard deviation of 2.3. An analysis on the factors affecting radon concentration such as age of the dwellings, floors, and stories, was performed. The mean effective dose to inhabitants has been calculated and found to be 8.2 mSv/y. Finally, for estimation of cancer risks, the lifetime risk and lung cancer cases per years per million have been calculated.

## 1. Introduction

^222^Ra and its progeny contribute more than half of human exposure from natural sources [1,2,3]. It is generated in rocks and soil by alpha decay of ^226^Ra and for this reason it is present on the entire Earth’s crust, although in varying quantities depending on the geology. After emission from the soil, radon can penetrate through cracks in walls and foundations inside homes where it can accumulate to harmful levels. Here, it decays and among its progenies, its short half-life alpha emitting daughters ^218^Po and ^214^Po contribute most to the dose. Being electrically charged, they can attach to dust or smoke particles in indoor air. During the breathing process, they can reach the bronchial tissue and there they decay emitting radioactive alpha particles capable of damaging the pulmonary epithelium and thereby causing lung cancer. UNSCEAR estimates an average annual effective dose to general population of 1.15 mSv/y attributable to inhalation of radon and its progenies. For this reason and also considering that people spend 80% of the time at home, many investigations of domestic radon exposure have been stimulated worldwide. In 2009, on the basis of recent epidemiological studies on both mine and dwelling exposures [4,5,6], the International Commission on Radiological Protection (ICRP 115) [7] has recommended a detriment-adjusted nominal risk coefficient for a population of all ages of 8 × 10^−10^ per Bq h·m^−3^ for exposure to ^222^Ra gas in equilibrium with its progeny (*i.e.*, 5 × 10^−4^ WLM^−1^). Moreover, a recent epidemiological study stated that indoor radon exposure might increase lung cancer risk in never-smokers [8]. It is well know that radon concentrations in homes depends on a number of parameters such as building materials, geology, living environment, climate and so on [9,10,11,12]. This also produces a large variability of concentrations of indoor radon on a small scale, to the point that rooms in buildings very close to each other or even in the same building may present very different radon concentrations. In Italy, only one national survey has been carried out that investigated the radon concentrations in dwellings in the early 1990s [13]. Subsequently, other studies have been performed on a local scale with different methods of measurement to determine the radon concentration in homes and workplaces in various Italian regions [14,15,16,17,18,19,20]. In this work we report the results of a radon survey conducted in 2013–2014 in 311 dwellings on the Peninsula Salentina of the Puglia region, in Southern Italy. This study area is located in the southern region of Puglia, between the Ionian Sea to the west and the Adriatic Sea to the east. Geologically, the area presents a training Cretaceous carbonate with lithological levels that consist of pure limestone and compact, sometimes slightly dolomite or limestone marl. The South Puglia region, although it can be considered as a low radon risk zone, presents important local anomalies that could be attributed to the presence of terra rossa that is the main source of ^222^Rn in the groundwater [21]. Moreover, Taroni *et al.* [22] showed that in the karst area of South Puglia the potential radon risk increased with the degree of alteration of subsoil structure; in particular in the zones characterized by high fracturing and big permeability, the range of radon in soil was high, from 400 Bq/m^3^ to over the 60 kBq/m^3^.

**Figure 1:**
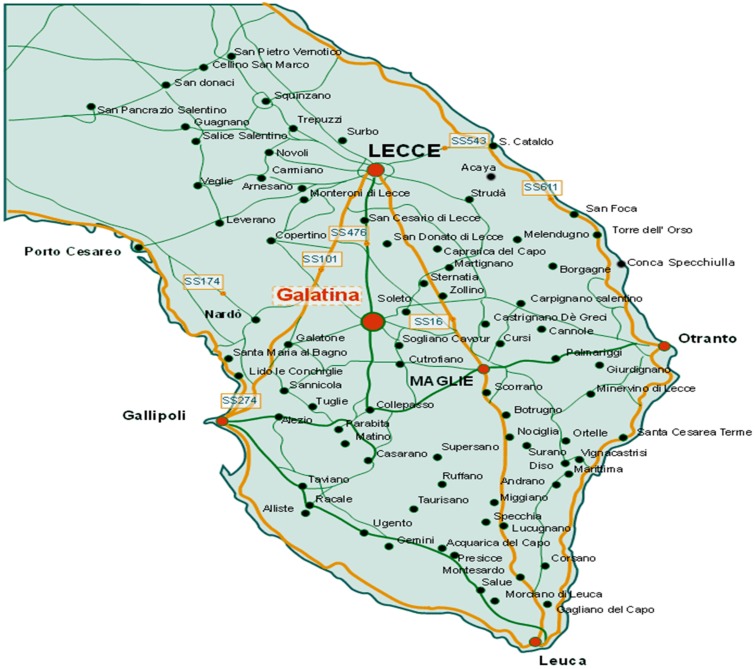
Map of Southern Puglia showing the area surveyed during the present investigation.

The municipalities involved in the survey were 16 (Sava, Cellino San Marco, Novoli, Ostuni, campi Salentina, Lecce, Trepuzzi, Sannicola, San Cesario, Copertino, Matino, Galatina, Racale, Carmiano, Specchia) and homes were selected in each of them. All data on measurement sites are reported in Figure 1 and on an interactive Google map where it is possible to see the data of every single measurement [23].

## 2. Materials and Methods

### 2.1. Radon Measurements

In September 2013 about 350 families were invited to participate in the indoor radon concentration survey. Each participant received a detector with instructions for its placement and a questionnaire concerning information about the main characteristics of their dwelling such as year of construction, main building materials, type and floor of the monitored room, *etc*. Unfortunately, due to mishandling by participants, 39 detectors were lost. The indoor radon concentration measurements were conducted using passive radon detectors, each equipped with a pair of LR-115 solid-state nuclear track detectors (SSNTD) provided by Dosirad (Pierrelatte, France). In each dwelling, the detectors was exposed in the rooms were the inhabitants spent the most of their time, generally the living room and bedroom, for six months. After exposure all detectors were chemically etched using a solution of 2.5 N NaOH at 60 °C for 110 min. For LR-115 detectors, the number of tracks increases linearly as the residual thickness decreases. In this study the residual thickness was measured with an optical method. The image of the detector was acquired by means of a scanner with double lighting and its mean brightness in the gray scale was determined using the image processing software ImageJ (Image Processing and Analysis in Java, version 1.46r, National Institutes of Health, (Bethesda, MD, USA). Using a previously determined calibration curve, the brightness was then converted into residual thickness. The automatic counting of tracks was also performed using the ImageJ software. The background track density was estimated to be 10 tracks/cm^2^ and it was determined counting the tracks of unexposed LR-115. Finally, the radon concentration was calculated using the Equation:

(1) $$C_{Rn}=\frac{N}{E\times T}$$

where *N* is the track density corrected by background track density and normalized to the nominal thickness of 6.5 μm, *E* is the efficiency and *T* is the exposure time.

### 2.2. Statistical Analysis

The geometric mean was used to describe the central tendency of the radon measurements because their distribution was skewed. No seasonal adjustment was applied to the radon concentration data. The normality of log-transformed data was tested by Shapiro-Wilk, and the homogeneity of variance was tested by Bartlett’s test. The comparison between two groups was performed with the non-parametric Mann-Whitney test for the log-transformed data. The comparisons between multiple groups were performed by non-parametric Kruskal-Wallis test. All statistical analyses were performed using the Stata software (Stata Corp., College Station, TX, USA).

## 3. Results and Discussion

### 3.1. Radon Measurements

**Figure 2:**
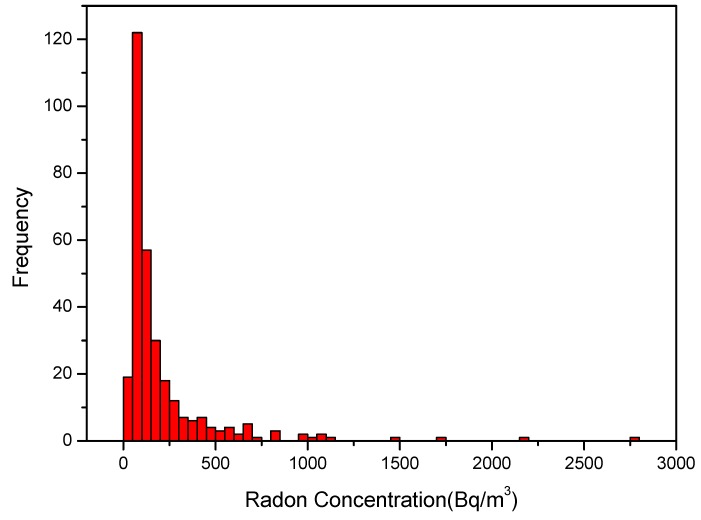
Frequency distribution of indoor radon concentrations in Peninsula Salentina dwellings.

The frequency distribution of the radon concentrations measured in 311 dwellings is shown in Figure 2. Although some authors [24,25,26] report that generally radon concentrations follow a log-normal distribution, the measured radon concentrations show an approximately log-normal distribution, although Shapiro-Wilk test failed to assess normality (*p* < 0.001). The reason for the lack of the log-normality could to be due to the great structural diversity of the geology of the sites and the variability of the types of housing. The minimum and maximum concentration were found to be 15 ± 2 Bq/m^3^ and 2166 ± 133 Bq/m^3^, respectively, with an geometric mean of 114 Bq/m^3^, geometric standard deviation of 2.3 and the median value is 104 Bq/m^3^. Overall, 74% of the dwellings presented radon concentrations lower than 200 Bq/m^3^, 9% had radon concentrations between 200 and 300 Bq/m^3^, and the other 17% had values greater than 300 Bq/m^3^.

**Figure 3:**
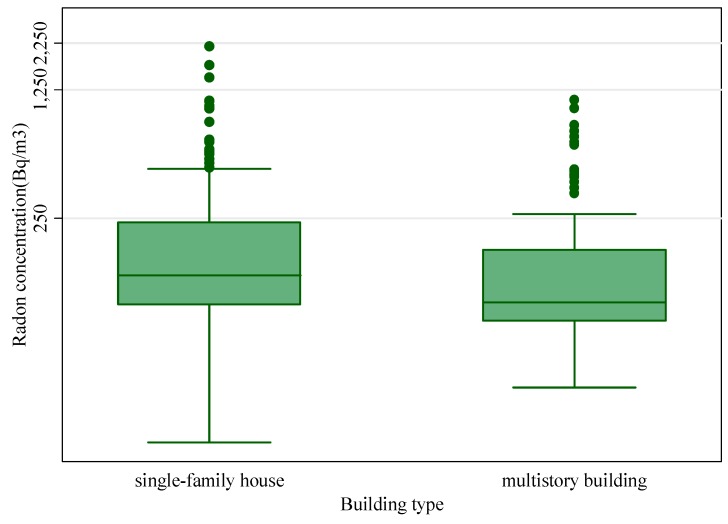
Relationship between radon concentrations and building types.

Overall, 65% of the monitored dwellings were single-family houses, while 35% were multi-storey buildings. Using the Mann-Whitney test, a significant statistical difference (*p* < 0.05) was found between median of concentrations measured in the two building types, was observed in Figure 3. This result is in agreement with a study by Barros-Dios *et al.* [27] who observed that the radon concentrations declined with the number of the stories of a building.

**Figure 4:**
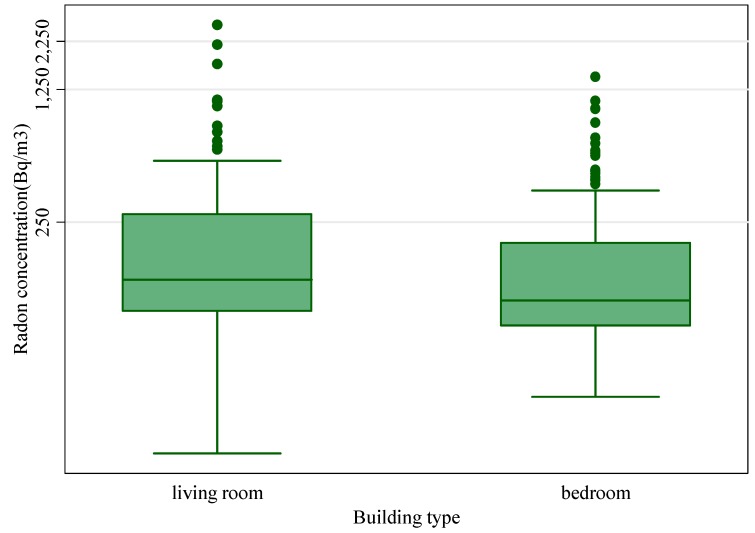
Relationship between radon concentrations and room types.

In 64% of the homes the detector was placed in the bedrooms, and a statistical significantly difference between the radon concentrations measured in the living room and bedroom was found (*p* < 0.05), with Figure 4 showing that the radon concentrations measured in the bedrooms are higher than those measured in living rooms. This difference could be attributed to minor opening of the windows during the night that contributes to increased indoor radon concentrations in the bedrooms. The finding of this study is in good agreement with the results from other authors [10,28] that found higher radon concentrations in bedrooms than in living rooms.

**Figure 5:**
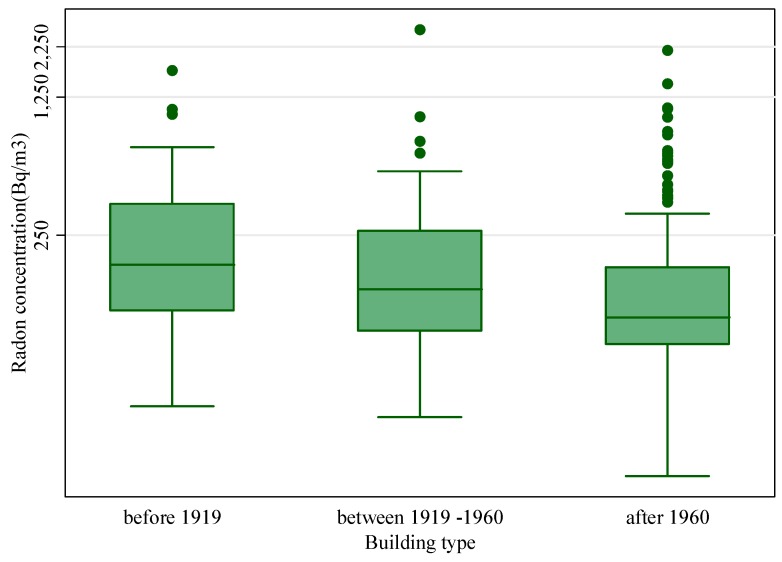
Relationship between radon concentrations and building age.

To evaluate the effect of the age of buildings on radon concentration, the houses were grouped in three categories: (i) built before of 1919; (ii) between 1919 and 1960 and (iii) after 1960 (Figure 5). The Kruskal-Wallis test showed that there is a significant difference (*p* < 0.05) in radon concentrations according to age of the dwellings. In particular, as shown in Figure 5 the median radon concentration is significantly higher for older houses compared with those built after 1960. Higher values reported for older houses could be explained by the fact that the ventilation rate in older houses is less than the new ones because they generally have smaller or fewer windows. Also older buildings have greater structural deterioration and poor ground insulation causing higher radon concentrations. Our results are consistent to that of other studies [27,29] that observed a statistically significant relationship between radon concentrations and dwelling age.

**Figure 6:**
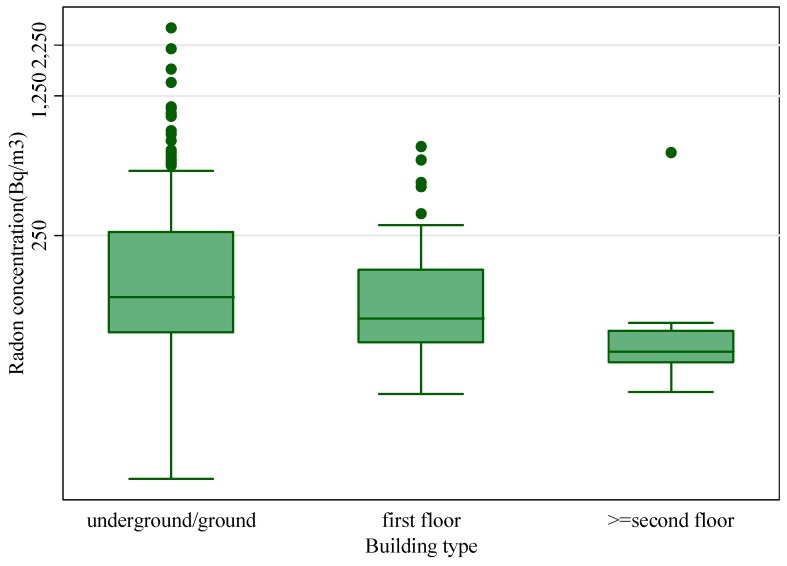
Relationship between radon concentrations and floor of the monitored rooms.

The statistical analysis showed that radon concentrations are significantly higher underground and at ground level compared with those of first and second floors (*p* < 0.01), as can be seen in Figure 6. This finding could be attributed to proximity of the underground and ground levels to the soil that represents the main source of indoor radon and also due to higher radon accumulation on lower floors. This dependence of the radon concentrations on floor level was observed also by other authors [10,11,27].

### 3.2. Annual Effective Dose

For the evaluation of the annual effective dose H to the inhabitants of the studied area due to the radon and its progeny, the UNSCEAR model was adopted, as reported in the following Equation:

(2) H(mSv/y) = C × F × O × T × D

where C stands for the average radon concentration equal to 209 Bq/m^3^, F is the equilibrium factor for indoor that is set as 0.4, O is the occupancy factor taken as 0.8, T is time in hours in a year (8760 h/y) and D is the dose conversion factor, 1.4 × 10^−8^ Sv per Bq/m^3^ h. The dose conversion coefficient reported in Equation (2) is obtained by equating the lung cancer risk of 8 × 10^−10^ per Bq/m^3^ h with total detriment for general population reported in ICRP 103 [29] that is 5.7 × 10^−2^·Sv^−1^. The mean annual effective dose was found to be 8.2 mSv/y.

Excess life time cancer risk (ELCR) was estimated using the following Equation:

(3) ELCR = H × DL × RF

where H is the mean effective dose, DL is the average duration of life estimated to a 70 years and RF is the fatal cancer risk per Sievert (5.5 × 10^−2^ Sv^−1^) recommended by ICRP 103. The mean excess lung cancer risk in the area was 3.1%. Finally, using the conversion ion factor for cancer cases per year per million per person of ICRP 50 [30] of 18 × 10^−6^ mSv^−1^·y, the radon-induced lung cancer risk was found to be 147.6 per million persons.

## 4. Conclusions

In this study, the results of a radon survey carried out in set of Peninsula Salentina (Southern Italy) dwellings are reported. Radon levels present a geometric mean of 114 Bq/m^3^ with a geometric standard deviation of 2.3. The findings show a statically significant correlation between indoor radon concentrations and some building characteristics such as floor level, age of the dwelling and number of stories. The estimated annual effective dose in the studied area was found to be 8.2 mSv/y. The radon-induced lung cancer risk presents a value of 147.6 per million persons.
